# Modulation of lower limb muscles and trajectory correction in the bipedal stance during visual perturbation

**DOI:** 10.7717/peerj.14631

**Published:** 2023-01-12

**Authors:** Tadayoshi Minamisawa, Noboru Chiba, Eizaburo Suzuki

**Affiliations:** 1 Department of Physical Therapy, Yamagata Prefectural University of Health Sciences, Yamagata, Japan; 2 Department of Occupational Therapy, Yamagata Prefectural University of Health Sciences, Yamagata, Japan

**Keywords:** Visual perturbation, Motor control, EMG–EMG coherence, Bipedal stance

## Abstract

The ability to actively track posture using visual targets as indicators is important for improving impairments in whole-body coordination, and accurate visual feedback on tasks is considered effective in promoting sensory-motor integration and behavioral success. In the present study, we examined inter- and intramuscular modulation between the two lower limbs in response to visual perturbation. Sixteen healthy young subjects (age: 21.3 ± 0.7 years) were asked to move their weight back and forth while tracking a visual target displayed on a monitor in front of them for 30 s. Three types of target movements were examined: a sinusoidal wave (*i.e.*, a predictable pattern), more complex patterns (random), and no movement (stationary). Electromyography (EMG) was used to assess intra- and intermuscular coherence modulation of the plantar flexor muscles (right and left soleus and right and left medial gastrocnemius). The ability to adjust posture to follow the target signal was assessed using a stabilometer. Inter- and intramuscular coherence increased during the visual perturbation task compared to the stationary task. In addition, left-right differences in lower limb modulation were observed during the visual perturbation task. Furthermore, interlimb coherence was related to the motor accuracy of tracking. The muscles of both lower limbs cooperated in response to visual perturbation, suggesting that these muscles control visually induced anteroposterior postural sway. Since such visual perturbations promote coordination between both lower extremities, this relationship may indicate the potential for rehabilitation training to help individuals acquire and improve the motor functions necessary to efficiently and stably perform activities of daily living.

## Introduction

Decreased ability to balance while standing is a serious problem in individuals with impaired motor function and may lead to falls (Berg & Cassells, 1992; Harris et al., 2005). Therefore, various methods have been developed to evaluate standing balance ability. Efficient approaches to understanding the neuromuscular mechanisms responsible for maintaining stability include external application of mechanical perturbations to increase the demands of sensory–motor integration and to induce specific modulations in the central nervous system (Munoz-Martel et al., 2019).

In particular, visually guided tracking tasks coupled with moving targets have been employed in rehabilitation to improve stationary and dynamic balance in individuals (Hung et al., 2014; Patel et al., 2011). However, use of regular or predictable task of visual targets has limitations, visuo-motor practice shifts from feedback to a predictive type of control after a few repetitions (Kitago & Krakauer, 2013). Therefore, tracking predictable targets (*e.g.*, targets following a sinusoidal wave pattern of movement) may not be useful for understanding neuromuscular performance or optimizing adaptive performance on tasks that require complex coordination such as posture and gait (Harrison & Stergiou, 2015; Stergiou & Decker, 2011). Because real-world environmental stimuli are unpredictable and complex, visual stimuli that induce feedback control of postural changes are important for improving motor processing performance (Hatzitaki et al., 2009). Thus, targets exhibiting motions with very complex patterns, such as mathematical chaos and randomness, have recently been employed in posture tracking research (Sotirakis et al., 2016; Sotirakis et al., 2017).

However, it remains unclear how humans process, integrate, and optimize visual information for postural control in tasks in which the target motion is completely random. In this study, we determined how lower limb muscle activity changes during visual perturbation. The cooperation of the plantar flexors during standing is thought to reflect an organizational relationship to maintain balance; indeed, a coherence analysis of electromyographic (EMG) data revealed the existence of an interaction network of muscle activity between the two lower limbs (Obata et al., 2014; Boonstra et al., 2015).

Inter- and intramuscular coherence (IMC) is an analysis of linear dependence between two EMG recordings at a certain frequency (Gross et al., 2002). This study aimed to answer the fundamental question of how the central nervous system organizes muscle activity in the lower limbs in response to visual information. Our findings may provide inform the restoration of physical function and optimization of quality of life in the future, including in individuals with impaired balance. In this study, we assessed whether coordination of lower limb muscles when actively tracking periodic or more complex movements of visual targets (*i.e.,* visual perturbation) confers a functional advantage. We hypothesized that more complex target movements would increase intermuscular coordination, especially in MG pairs, and that such intermuscular modulation would be associated with tracking accuracy relative to target motion. If such differences are observed, they will highlight the importance of bipedal gait control in daily life, which includes unexpected environmental disturbances.

## Materials & Methods

### Subjects

We recruited healthy young adults (six males, 11 females; age: 21.3 ± 0.7 years; height: 165.5 ±  7.0 cm; weight: 54.3 ±  4.6 kg) with no history of neuromuscular or skeletal diseases from a college population. All participation were provided with written information concerning the study and written consent was obtained. The experiment was approved by the Ethics Committee of Yamagata University of Health Sciences (approval ID: 2203-35) and was conducted in accordance with the Declaration of Helsinki.

### Measurement tasks and content

Participants were instructed to stand barefoot and with their eyes open, with each foot on a separate stabilometer (20-Hz sampling frequency, G-6100; ANIMA; Japan), and to maintain comfortable arm and leg widths.

**Figure 1:**
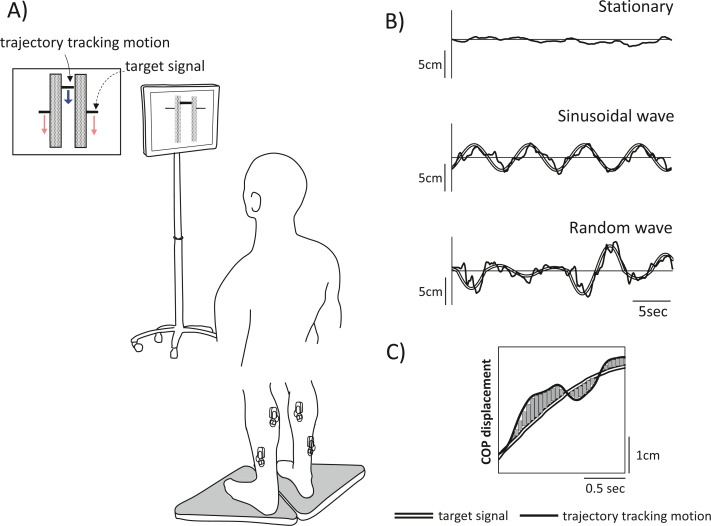
Experimental schematic and tracking task used in the experiment. (A) Experimental setup. Participants were instructed to stand on two stabilometers while tracking a vertically moving visual target (dashed arrow). Movement was represented by forwards or backwards shifts of the centre of pressure (CoP); anterior shifts of the CoP correspond to upwards movement, and posterior shifts of the CoP correspond to downwards movement. Each tracking task lasted 30 s, and there were three tracking tasks in total: two visual perturbation tasks with different target movement (periodic [sinusoidal] *vs.* non-periodic [random]) and a stationary standing task as a control. (B) The tracking task used in the experiment. Top row, stationary; middle row, sinusoidal movement, and bottom row, random movement. The double line shows the computer-generated target signal, and the solid line shows the trajectory of the participant tracking motion. (C) To clarify the tracking accuracy, the displacement of the trajectory of participant motion relative to the target signal motion during visual perturbation was calculated as the sum of the areas. The figure shows a partial time-series waveform.

The participants performed a tracking task in which they were instructed to follow two visual, vertically oscillating targets displayed on a monitor screen (42 × 24 cm; placed 1 m in front of them) by rocking their bodies back and forth without bending their hips or knees (Fig. 1). We referred to previous published reports to three tracking tasks (stationary *vs.* sinusoidal *vs.* random waveform) (Sotirakis et al., 2017). The bipedal stance (*i.e.,* the stationary standing posture) was also measured as a control task. EMG signals were obtained using a wireless EMG system (BTS-FREEEMG (BTS-FREEEMG1000; BTS Bioengineering, Milan, Italy)) from muscles in both lower limbs: the medial gastrocnemius muscles (right: MGr, left: MGl) and the soleus (right: SOLr, left: SOLl). Prior to placement of the electrodes to collect the EMG data, the skin of participants was washed with alcohol solution and carefully prepared to ensure good electrode contact. EMG was recorded at a distance of 2.5 cm between electrodes. Signals were preamplified (gain: 2,000, bandwidth: 20–500 Hz) and transmitted with a sampling frequency of 1,000 Hz and a 16-bit resolution. To determine whether inter- and intralimb muscle activity in a task was associated with balance, the variability in the centre of pressure (CoP) along the anteroposterior axis for each limb was measured by the stabilometers. These individual CoP measurements (*i.e.,* one for each limb) were used to calculate the composite CoP in the anteroposterior direction (CoPap) for subsequent analyses. The centre of pressure (CoP) is the point given to the point of application of the ground reaction force vector. This stabilometers consists of three load cells and calculates the CoP from the vertical component of the ground reaction force. Digital signals from the two stabilometers were manually synchronized and stored on a personal computer for further processing.

### Target motion

In the visual perturbation task, the bar indicating the trajectory of subject tracking motion was displayed in the centre of the screen, and the targets were displayed at either end of the bar. The targets moved up and down on the screen, cueing participants to move forwards and backwards, respectively. Upwards movement of the target corresponded to forwards shifts in the CoP of subjects, while downwards movement of the target corresponded to backwards shifts in the CoP of subjects. The oscillation of the target movement had a waveform with a frequency of 0.13 Hz, following the methods of a previous study (Patikas et al., 2019). Two types of waveforms were used: a sinusoidal waveform and a random waveform. The midpoint of the CoPap during the stationary standing task was used as the reference point for amplitude generation, and the magnitude of the amplitude of the sinusoidal waveform in the anteroposterior direction was ±3 cm. The random waveform was programmed to add to or subtract three cm from the sinusoidal waveform and thus further increase or decrease the amplitude in a random manner. This target movement pattern not only constantly fluctuated but also included zero amplitudes, where the target did not cue any participant movement. Three trials of each of three postural tasks (stationary standing, two visual perturbation tasks) were performed in a pseudo-randomized order for a total of nine trials; each trial lasted 30 s with a 1-minute rest between trials, and one practice trial was administered before the experiment began.

### Data processing and analysis

To estimate EMG coherence of muscle pairs, EMG signals were high-pass filtered at a frequency of 20 Hz using a fourth-order Butterworth filter with zero phase delay and rectified with a Hilbert transform (Boonstra et al., 2015). The power spectrum of the envelope extracted EMG signal is widely used as a method to enhance firing rate information (Myers et al., 2003). The power spectral density of the EMG envelope and EMG coherence were estimated using Welch’s method with muscle pairs concatenated with a window length of 2,500 points and 50% overlap. The correlation between muscle activity modulations of both lower limbs was analysed. Spectral coherence, the common oscillation of the two EMG signals, was estimated in the frequency domain from 0 to 10 Hz (in 2-Hz bands) for the four muscle pairs: (1) the bilateral SOL (SOLr–SOLl), (2) the bilateral MG (MGr–MGl), (3) unilateral (right) SOL and MG (SOLr–MGr), and (4) unilateral (left) SOL and MG (SOLl–MGl) pairs. Coherence of these four pairs were compared between tasks. These data analyses were performed using NI DIAdem 2020 (National Instrument, Austin, TX, USA). In this study, we also determined the confidence level (CL) of coherence; a coherence value is considered significant if the CL is above a certain level (Rosenberg et al., 1989). Coherence values above the 95% confidence limit were considered significant. In this study, the coherence threshold was set at 0.063. To reveal the temporal synchrony between muscle pairs, phase angles were calculated from the complex valued cross power spectral density. The CoP signal was filtered with a fourth-order Butterworth low-pass filter with a cut-off frequency of 20 Hz. To clarify participant performance on the visual perturbation tasks, the discrepancy between the CoPap tracking and the target signal (sinusoidal and random waveforms) was calculated as a trapezoidal area over the entire measurement time to obtain the sum of area (SoA). If there was a point discrepancy between the coordinates of the two curves, the area was calculated after generating the points by interpolation.

### Statistical analysis

Posteriory analysis with G*Power (Faul et al., 2009) showed that with a sample size of 16, effect size of 0.46 and probability level of 0.05, the resulting power estimate was 0.96. First, a Shapiro Wilk test was used to test the normality of the data (*p* > 0.05). A two-way repeated-measures ANOVA was performed to determine how EMG coherence differed according to task and phase. In particular, we focused on the effect of task (stationary *vs.* sinusoidal *vs.* random waveform) because we were interested in muscle modulation due to visual perturbations. If an effect was significant, we performed post hoc paired t tests with a Bonferroni correction. Mann–Whitney *U* test or Student’s paired *t*-test were performed for the MGr–SOLr and MGl–SOLl muscle pairs to compare the intralimb coherence in response to visual perturbation. The statistical significance level was set at *p* < 0.05. The data presented are the task means and standard deviations. The Spearman’s correlation analyses were used to analyse the relationship between coherence and the SoA in each frequency band of the muscle pairs to determine the relationship between the muscle activity modulation and tracking accuracy. The strength of the relationship between the SoA and coherence was defined as low if the correlation coefficient (*r*) was between 0.1 and 0.3, medium if it was between 0.3 and 0.5, and high if it was greater than 0.5. All statistical analyses were performed using OriginPro 2017 (OriginLab, Northampton, MA, USA).

## Results

The presents study examined the modulation of lower limb muscle activity when healthy young adults were subjected to a visual perturbation task. Furthermore, we analysed the correlation coefficient between the coherence values of such plantar flexor muscle pairs and the CoP, which reflects the ability to regulate posture.

### Inter- and intramuscular coherence analysis

**Figure 2:**
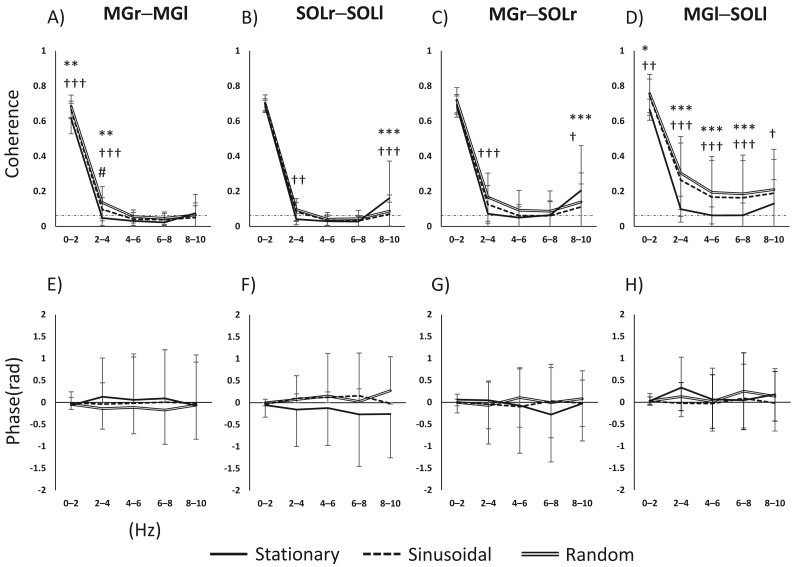
Results of a two-way repeated-measures ANOVA of the changes in activity in the four muscle pairs (in the 0–10-Hz bands) on three tasks. (A–D) The results of coherence analysis, and (E–H) the results of phase analysis. The line patterns (solid, dashed, and double) represent stationary, sinusoidal movement, and random movement, respectively. *, stationary *vs.* sinusoidal; †, stationary *vs.* random; #, sinusoidal *vs.* random. The number of asterisks indicates the level of importance. For example, asterisks (*, **, and ****) indicate *p* < 0.05, *p* < 0.01, and *p* < 0.001, respectively. Abbreviations: MG, medial gastrocnemius; SOL, soleus; r, right limb; l, left limb.

The main effects and interactions in muscle pair and frequency for task and frequency were as follows. For the MGr–MGl pair, there was a significant main effect of task (*F*(2, 98) = 15.669, *p* < 0.001), frequency (*F*(4, 196) = 3136.2555, *p* < 0.001) and the task × frequency interaction (*F*(8, 392) = 10.331, *p* < 0.001). For the SOLr–SOLl pair, the main effect of task was not significant (*F*(2, 98) = 2.161, *p* = 0.121); the main effect of frequency was significant (*F*(4, 196) = 1597.584, *p* < 0.001), and the task × frequency interaction was significant (*F*(8, 392) = 12.882, *p* < 0.001). For the MGr–SOLl pair, the main effect of task was not significant (*F*(2, 92) = 2.620, *p* = 0.078), the main effect of frequency was significant (*F*(4, 184) = 1044.013, *p* < 0.001), and the task × frequency interaction was significant (*F*(8, 368) = 11.311, *p* < 0.001). For the MGl–SOLr pair, the main effect of task was significant (*F*(2, 94) = 21.847, *p* < 0.001), the main effect of frequency was significant (*F*(4, 188) = 902.02, *p* < 0.001), and the task × frequency interaction was significant (*F*(8, 376) = 7.858, *p* < 0.001). For both interlimb MG and SOL pairs, coherence was higher in the 0–4 Hz band in the visual perturbation task compared to the stationary standing task (Figs. 2A and 2B). The intralimb MGr–SOLr showed increased coherence during the visual perturbation task compared to the stationary standing task. The intralimb MGl–SOLl pair showed a significant increase in the coherence of all frequency bands (0–10 Hz) during the visual perturbation task compared to stationary standing task (Figs. 2C and 2D).

### Inter- and intramuscular phase

Since the inter- and intramuscular coherence was found to be partially statistically significant, a subsequent analysis was performed using phase analysis to reveal the temporal delay of the muscle pairs. The main effects and interactions of task and phase in the muscle pair and frequency band were as follows. For the MGr–MGl pair, the main effect of task was not significant (*F*(2, 64) = 2.855, *p* = 0.065), the main effect of phase was not significant (*F*(4, 128) = 0.052, *p* = 0.995), and the task × phase interaction was not significant (*F*(8, 256) = 0.711, *p* = 0.682). For the SOLr–SOLl pair, the main effect of task was significant (*F*(2, 64) = 7.722, *p* < 0.001), the main effect of phase was not significant (*F*(4, 128) = 0.147, *p* = 0.964), and the task × phase interaction was not significant (*F*(8, 256) = 1.643, *p* = 0.113). For the MGr–SOLl pair, the main effect of task was not significant (*F*(2, 64) = 0.992, *p* = 0.377), the main effect of phase was not significant (*F*(4, 128) = 0.072, *p* = 0.990), and the task × phase interaction was not significant (*F*(8, 256) = 1.382, *p* = 0.205). For the MGl–SOLr pair, the main effect of task was not significant (*F*(2, 64) = 0.915, *p* = 0.406), the main effect of phase was significant (*F*(4, 128) = 2.532, *p* = 0.044), and the task × phase interaction was not significant (*F*(8, 256) = 0.790, *p* = 0.611). Figures 2E–2H show the phase-frequency plots of the muscle pairs. The phase-frequency plots were nearly flat and approximately 0 between all muscle pairs. There were no significant differences in the phase-frequency relationship between the stationary standing task and the two visual perturbation tasks. When the phase difference between paired muscle activities is zero, such muscle pairs will have the same sign and will reinforce each other. Thus, it is clear that in three tasks, both lower limbs respond to each other’s task without any time difference.

### Comparison for intramuscular coherence in the left and right lower limbs

**Figure 3:**
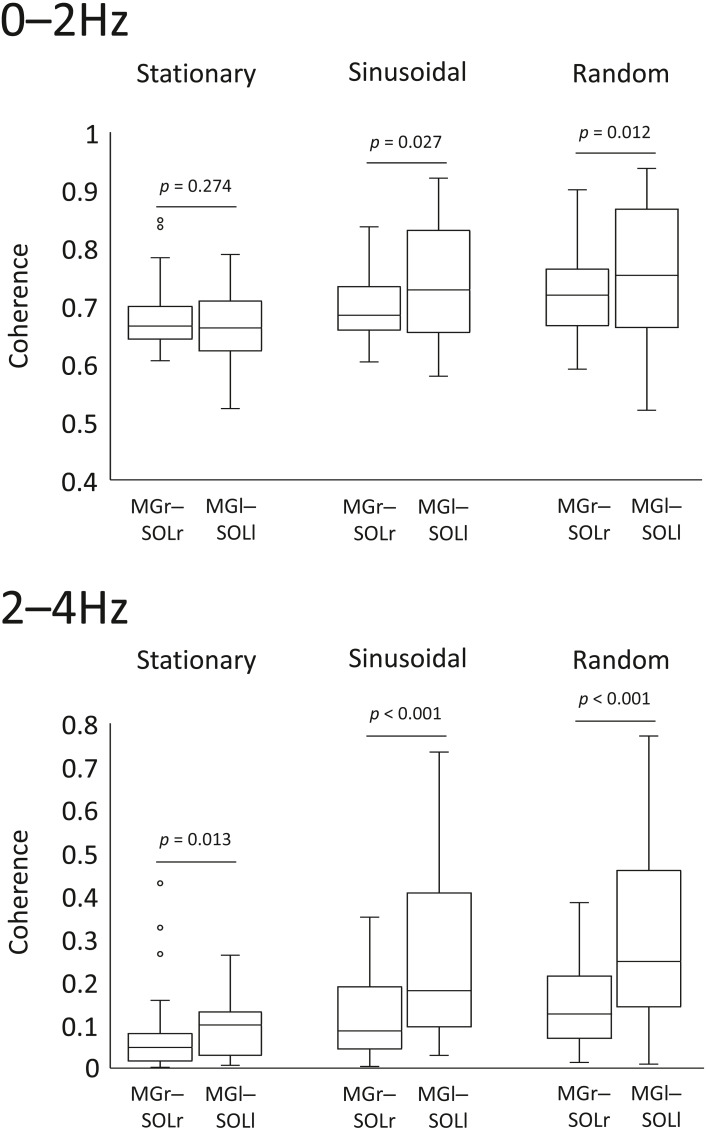
Comparison of coherence of muscle pairs in the left and right lower limbs in each task. Coherence of the muscle pairs of the right and left lower limbs in each task (*i.e.,* right lower limb: gastrocnemius-soleus muscle pair *vs.* left lower limb: gastrocnemius-soleus muscle pair) were analysed. The lower and upper whiskers of the boxplot represent the minimum value within 1.5 interquartile range (IQR) of the first quartile and the maximal value within 1.5 IQR of the third quartile value, respectively, and the white circle marks the outliers.

In the sinusoidal and random visual perturbation tasks, the 0–2 Hz and 2–4 Hz bands exhibited statistically significant differences in coherence between the MGr–SOLr and MGl–SOLl pairs (Fig. 3). In the stationary standing task, there was no significant difference in coherence between the left and right lower limb muscles for the 0–2 Hz band, but there was a significant difference for the 2–4 Hz band. These data indicate that the modulation of the left and right lower limb muscles is not necessarily identical.

### Correlation analysis between coherence and the sum of area

**Figure 4:**
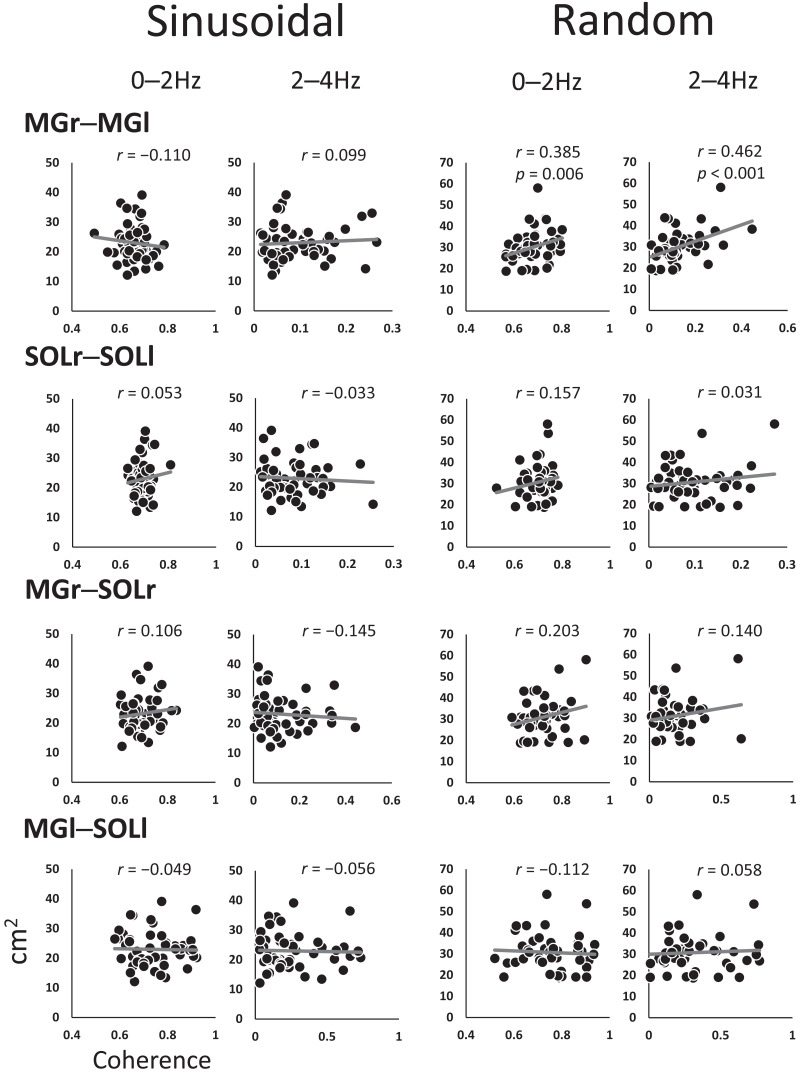
Scatter plot of the correlation between the coherence and sum of areas for each muscle pair. The analysis was restricted to the frequency bands of 0–2 Hz and 2–4 Hz, where coherence was high, with reference to the confidence level (CL) of the coherence values. The vertical axis indicates the sum of the area, and the horizontal axis indicates the coherence. Abbreviations: MG, medial gastrocnemius; SOL, soleus; r, right limb; l, left limb.

Figure 4 shows the coefficients of the correlations between the SoA and coherence for each muscle pair at every 2-Hz band. The 0–2 Hz (*r* = 0.385, *p* = 0.006) and 2–4 Hz (*r* = 0.462, *p* < 0.001) bands for the MGr–MGl muscle pair in the random visual perturbation tasks showed a moderate correlation between coherence and the SoA. No significant correlations between coherence and the SoA were found in the frequency bands of the other muscle pairs.

## Discussion

In the present study, we investigated inter- and intramuscular modulation of lower limb muscles in response to visual perturbation during bipedal standing, furthermore, the ability to control standing during perturbation. The results showed a significant increase in coherence values of muscle pairs under visual perturbation stimulation conditions compared to resting standing, and a significant difference when comparing unilateral heteronymous muscle pairs in the left and right lower limbs. In addition, there was a relationship between the coherence of such muscle pairs and the ability to control the standing posture. These findings contribute to our understanding of neuromuscular control in response to perturbing stimuli, particularly because they reveal modulations in central neural processing with respect to control of homologous muscles in response to visual perturbations. These findings are discussed in detail below.

### Intermuscular coherence during visual tracking tasks

First, we assessed intermuscular coherence during visual tracking tasks to determine the extent of coordination between the lower limbs We found that interlimb MG and SOL pairs (*i.e.,* MGr–MGl and SOLr–SOLl) organized interlimb control by increasing modulation in the low-frequency band to cope with visual perturbations. In particular, the interlimb MG pair showed significantly increased coherence in the 2–4-Hz band in response to random waveforms compared to sinusoidal waveforms or stationary visual stimuli. These changes are similar to previous studies examining muscle modulation with unstable surfaces as co-activation increase with increasing difficulty of the postural task (Azizi, Brainerd & Roberts, 2008) and may act as a compensatory mechanism by increasing joint stiffness and thereby stability (Patikas et al., 2019). Both the MG and SOL are muscles that cooperate during standing, and both plantar flexors synergistically regulate ankle joint torque and contribute to the maintenance of balance (Obata et al., 2014). Interlimb coordination is necessary to maintain the centre of mass (CoM) in the bipedal stance (Genthon & Rougier, 2003; Dietz, Horstmann & Berger, 1989); thus, the present results indicate that interlimb coordination by muscle modulation is important for humans to maintain a bipedal stance during visual perturbation.

### Intramuscular coherence during visual tracking task

There was a significant increase in coherence in the 2–4-Hz band for the right lower limb and a significant increase in coherence in the 0–10-Hz bands in the left lower limb during the visual perturbation tasks compared to stationary standing. Performing a tracking task while standing requires not only the ability to track the target but also the ability to compensate for visual perturbation and maintain balance. If visual perturbations disrupt the visuomotor integration in the bipedal stance (Sotirakis et al., 2016), increases in the broadband modulation of the left lower limb may reflect a strategy to stabilize motion by employing muscle modulation (Latash, 2018). On the other hand, differences in coherence during visual perturbation were observed in the 2–4-Hz and 8–10-Hz bands in the unilateral heteronymous muscles of the right lower limb (*i.e.,* the MGr–SOLr pair). The lower coherence in the 2–4-Hz band may result from the need to respond quickly and accurately to complex visuomotor cues in the visual perturbation task (Gatev et al., 1999; Masani et al., 2003).

We argue that the aforementioned differences in muscle modulation of the left and right lower limbs in response to visual perturbation suggest differences between the functions of the lower limbs during standing.

### Correlation analysis of inter- and intramuscular coherence and SoA

Interlimb coordination by the MG pair (*i.e.,* the MGr–MGl pair) was associated with increased accuracy of target signal tracking, and moderate correlations were observed between intermuscular coherence and the SoA. In general, the MG and other plantar flexors during postural sway compensate for induced perturbation (Héroux et al., 2014). Since fine-grained modulations of muscle pair coherence improve tracking accuracy (*i.e.,* reduce the SoA) on tracking tasks compared to coarse-grained modulations, it may be functionally preferable for each lower limb to respond to perturbations with different stabilizing or propulsive roles, rather than robust synchronization by both muscle pairs.

Understanding and investigating inter- and intralimb coordination in response to visual perturbation may help optimize functional recovery and quality of life of patients. In addition, acyclic tracking tasks (*i.e.,* random target motion) may be implemented in neurological exercise programs to facilitate feedback–based control, improve internal models of postural motor commands and subsequent sensory consequences, and prevent future falls (Sotirakis et al., 2019). In other words, if such visual perturbations promote interlimb coupling, this relationship will indicate the possibility of rehabilitation training for individuals to acquire and enhance the motor function necessary for efficient and stable performance of tasks and activities of daily living. In the future, additional experimental approaches targeting various levels of motor function may provide further insight into the significance of EMG-EMG coherence changes associated with declining physical function.

There are several limitations to this study. Although the present study used the SoA as a measure of sway accuracy in the tracking task, the random stimuli had different trajectory lengths across trials. It is unclear how such differences affect coherence. One solution might be adaptable to use Mean Squared Error and normalize it to the area due to the difference between the actual COP trajectory and the target signal, divided by the distance. Second, we assessed only the plantar flexor muscles in this study. Participants may have also attempted to respond to visual perturbations by firmly immobilizing the ankle joint; therefore, the association of visual perturbation with activity of the tibialis anterior, the antagonist muscle of the MG and SOL, may be needed to evaluate functional significance.

## Conclusions

The results of this study suggest that muscle modulation is greater when tracking random target motion, and the effect of muscle modulation on coherence depends on target motion complexity. Additionally, tracking accuracy and muscle pair modulation were positively correlated. In the future, larger sample sizes may provide an opportunity to increase the precision of the study and significantly improve our understanding of the modulation of intermuscular networks in response to visual perturbation stimuli.

## Additional Information and Declarations

### Data Availability

The following information was supplied regarding data availability: The data is available at the Open Science Framework: Minamisawa, Tadayoshi. 2022. “Modulation of Lower Limb Muscles and Trajectory Correction in the Bipedal Stance during Visual Perturbation”. OSF. September 10. osf.io/v45bw.
